# Use of Botanical Supplements Among Romanian Individuals with Diabetes: Results from an Online Study on Prevalence, Practices, and Glycemic Control

**DOI:** 10.3390/nu17152440

**Published:** 2025-07-25

**Authors:** Cosmin Mihai Vesa, Delia Mirea Tit, Emilia Elena Babes, Gabriela Bungau, Andrei-Flavius Radu, Radu Dumitru Moleriu

**Affiliations:** 1 Doctoral School of Biomedical Sciences, Faculty of Medicine and Pharmacy, University of Oradea, 410087 Oradea, Romania; cosmin.vesa@csud.uoradea.ro (C.M.V.); gbungau@uoradea.ro (G.B.); andreiflavius.radu@uoradea.ro (A.-F.R.); 2 Department of Preclinical Disciplines, Faculty of Medicine and Pharmacy, University of Oradea, 410073 Oradea, Romania; 3 Department of Pharmacy, Faculty of Medicine and Pharmacy, University of Oradea, 410028 Oradea, Romania; 4 Department of Medical Disciplines, Faculty of Medicine and Pharmacy, University of Oradea, 410073 Oradea, Romania; 5 Department of Psicho-Neurosciences and Recovery, Faculty of Medicine and Pharmacy, University of Oradea, 410073 Oradea, Romania; 6 Department III of Functional Sciences, “Victor Babes” University of Medicine and Pharmacy, 300041 Timisoara, Romania; radu.moleriu@umft.ro

**Keywords:** diabetes mellitus, plant extracts, natural medicine, complementary medicine, glycemic control

## Abstract

Plant supplements are frequently used by diabetes mellitus (DM) patients in the management of their disease. **Background/Objectives:** The present study aimed to identify the prevalence of plant supplement use in DM patients from Romania and to evaluate patients’ practices, profiles, and beliefs regarding plant supplements and the impact of their use on glycemic control. **Methods:** A cross-sectional online survey was conducted among Romanian diabetic patients. **Results:** Out of 329 validated responses, 44.07% reported supplement use. *Momordica charantia* L. (35.17%) was the most used. Female patients were statistically significantly more prevalent in the plant supplement user group. Plant supplement use was associated with statistically significantly lower HbA1c (7.11% vs. 7.66%, *p* < 0.01) and basal glycemia (127.75 mg/dL vs. 136.08 mg/dL, *p* < 0.01) over the previous three months. Diabetic polyneuropathy was statistically significantly less prevalent among patients who used plant supplements (31.03% vs. 42.39%, *p* = 0.035). The greatest proportion of responders reported that they started to use plant supplements for improving blood glycemia (88.97%), followed by the purpose of preventing DM complications (27.59%). A significant improvement in health status was reported by 53.79% of patients using herbal supplements. **Conclusions:** Plant supplement use was common and associated with improved glycemic parameters and lower complication prevalence.

## 1. Introduction

Diabetes mellitus (DM) comprises a group of metabolic disorders characterized by chronically elevated blood glucose levels due to insufficient insulin production, impaired insulin action, or a combination of both [1]. Poor glycemic control significantly increases the risk of serious complications, including cardiovascular disease, kidney damage, neuropathy, retinopathy, and even lower-limb amputations [2]. Globally, the burden of diabetes has escalated dramatically, with the number of affected individuals rising from 211.2 million in 1990 to 476 million in 2017 [3].

While multiple pharmacological therapies are available for diabetes management, many come with limitations. Metformin, the standard first-line treatment for type 2 diabetes, is widely prescribed due to its affordability, long-term glycemic benefits, and favorable cardiovascular profile. However, gastrointestinal side effects can render it intolerable for some patients [4]. Other medications, such as sulfonylureas, are effective at lowering blood glucose by stimulating insulin release but are associated with risks like weight gain and hypoglycemia, especially in older adults with compromised renal function [5]. More recent drug classes, including DPP-4 inhibitors, GLP-1 receptor agonists, and SGLT-2 inhibitors, offer advantages such as cardiovascular protection, weight neutrality, and improved HbA1c levels. Nonetheless, these therapies may still be accompanied by side effects such as gastrointestinal distress, urinary tract infections, or rare risks like pancreatitis [6,7,8].

Considering the above challenges, traditional and complementary therapies, particularly plant-based compounds and supplements, are gaining traction as supportive or alternative strategies in diabetes management [9]. Rooted in centuries-old cultural and healing practices [10], these remedies often feature bioactive compounds such as flavonoids, alkaloids, polyphenols, and tannins. These natural constituents may contribute to better glycemic control by enhancing insulin secretion, improving glucose uptake, inhibiting hepatic glucose production, or reducing intestinal glucose absorption. They may also exert anti-inflammatory and antioxidant effects [11,12,13]. As such, functional foods and phytotherapies are increasingly recognized for their potential to improve diabetes outcomes with fewer side effects compared to conventional drugs [14,15].

We investigated the use of plant-based supplements among Romanian patients with DM. Specifically, our research sought to determine the prevalence of use, the types of supplements commonly consumed, and their self-reported impact on glycemic control and diabetes-related complications. Additionally, we explored the motivations, beliefs, and decision-making factors that guide patients’ choices regarding phytotherapy. To achieve these objectives, we conducted a nationwide cross-sectional survey, disseminated via diabetes-related social media platforms, offering unique insights into how plant-based therapies are integrated into everyday diabetes self-management practices in Romania.

## 2. Results

### 2.1. General Information

Table 1 presents the socio-demographic and clinical characteristics of participants based on their use of plant supplements. Among the 329 validated responses, 145 participants (44.07%) reported using plant supplements (YES group), while 184 participants (55.93%) did not use plant supplements (NO group). Female participants were significantly more likely to use plant supplements compared to males (68.28% vs. 55.43%, *p* = 0.018). No significant differences were observed between the groups in terms of age (median 62 [IQR 16] vs. 59.5 [IQR 16.3] years, *p* = 0.068) or diabetes duration (median 5 [IQR 10] vs. 8 [IQR 11] years, *p* = 0.068), nor in education level distribution (*p* = 0.291). Regarding treatment, basal insulin use was significantly more common in the NO group (31.52%) compared to the YES group (19.31%) (*p* = 0.027), while the proportion of participants not taking any medication was significantly higher in the YES group (5.52% vs. 1.09%, *p* = 0.020). Other treatments (e.g., biguanides, sulfonylureas, GLP-1 agonists) showed no significant differences between the groups.

**Figure 1:**
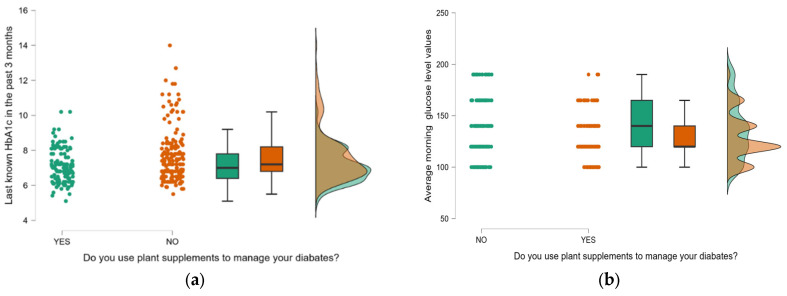
Distribution of glycemic parameters according to plant supplement use. (**a**) HbA1c, (**b**) basal glucose. YES, those using plant supplements; NO, those not using plant supplements.

The analysis of HbA1c and basal glucose levels shows that individuals using plant supplements had better glycemic control compared to non-users. The median HbA1c values were lower in supplement users (7.00%) compared to non-users (7.20%). Similarly, the mean HbA1c was lower in the supplement group (7.11%) than in non-users (7.66%) with a statistically significant difference (*p* = 0.001) and a narrower confidence interval, suggesting more consistent glycemic control (Figure 1a). Basal glucose levels followed a similar trend, with supplement users having a lower median (120.00 mg/dL) compared to non-users (140.00 mg/dL) and a statistically significant mean difference (127.75 mg/dL vs. 136.08 mg/dL, *p* = 0.009) (Figure 1b). Variability in glucose control, indicated by standard deviation and interquartile range, was lower in supplement users, reflecting more stable glycemic levels.

A higher percentage of plant supplement users (41.38%) achieved glucose levels in the 110–130 mg/dL range compared to non-users (25.54%), while fewer plant supplement users (2.07%) had dangerously high levels (> 180 mg/dL) compared to non-users (10.33%) (Table 2).

To straighten the statistical analysis and to highlight the main conclusion that the consumption of natural supplements significantly influences the health of DM patients, a risk analysis was run. The use of supplements was considered as a protective factor; a value bigger or equal to 6.5% for HbA1c or a basal glycemia value bigger or equal to 100 mg/dL was considered abnormal. In both cases, a significant protective factor was obtained. Because in both cases the *p* value was below the cutoff point, it can be concluded that the protection given by the studied supplements is significant for the whole population (Table 3).

Regarding the prevalence of diabetes-specific complications, where glucose control is a critical factor, diabetic polyneuropathy was significantly less prevalent in the group using plant supplements compared to the non-users (31.03% vs. 42.93%, *p* = 0.0035). However, no significant differences were observed between the groups for chronic kidney disease (*p* = 0.395) or retinopathy (*p* = 0.884) (Table 4).

### 2.2. Plant Supplement Use

*Momordica charantia* L.-based plant supplements were the most frequently used (35.17%) among the plant supplement users, followed by plant combination supplements (15.86%), blueberry-based supplements (15.17%), and blackseed-based supplements (7.59%) (Table 5). Resveratrol (0.69%), spirulina (1.38%), evening primrose (0.69%), and cinnamon (0.69%) plant supplements were rarely used by diabetes patients.

The content of the plant combination supplements used by the diabetes patients in our study is detailed in Table 6. It can be observed that *Momordica charantia* L. is present in almost all types of plant combination supplement for DM management.

The plant user group consumed the supplements mainly as capsules (65.52%) or tablets (17.93%), while teas and tinctures represented the less used pharmaceutical forms, indicating the patients’ need for easily administered supplements that are not time consuming to prepare (Table 7).

Improvement of blood glucose levels was the most frequent reason (88.97%) for starting use of plant supplements, while 27.59% of patients responded that they started using the supplements for preventing diabetes complications. Of the patients surveyed, 69.66% continued to use them because they trusted their effect. It is worth observing that plant supplement use was recommended by health professionals in 46.90% of cases, including doctors (26.90%) and pharmacists (20%), and that friends or mass media rarely influenced this decision. Many patients took plant supplements on their own initiative (40.69%), a fact that might suggest the presence of popular belief in the benefits of plant supplements in disease management. A total of 53.79% of patients observed significant improvement in their health, a fact that suggests that the objective improvement of certain parameters could be documented by the patients (Table 8).

An improvement in basal glycemia was the most frequent parameter of improvement. Diabetes patients most frequently monitor their disease by measuring blood glucose in the morning. Therefore, it is not surprising that other parameters such as postprandial glycemia, body weight, or cholesterol values were not identified frequently as parameters that improved after plant supplement use because they are not measured as often as basal glycemia (Table 9).

When patients not using plants supplements were asked about their reasons for not using them, 43.38% responded that they did not trust their effect, while 38.04% responded that they did not use them because their doctor had not recommended them (Table 10).

## 3. Discussion

Complementary and alternative medicine has grown in popularity as a treatment for DM, due to its low risk of complications and low expense. Additionally, complementary and alternative medicine has emerged as a viable option when considering the cultural and psychosocial factors, health beliefs, and religious values [16]. Recent research has demonstrated that plant-based formulations, in conjunction with a carefully thought-out diet, can help improve diabetes management because specific phytochemicals have anti-inflammatory, antioxidant, and glucose-lowering properties [17,18]. The use of plant supplements is encountered not only in societies under development but also in Europe: a survey conducted on the general population concluded that 18.8% of the participants who were screened reported using at least one plant food supplement [19]. The percentage of the Romanian population in the same survey using plant supplements was 17.6% [19].

Much higher percentages of individuals taking plant food supplements have been identified in populations with chronic diseases such as DM, where achieving certain medical targets regarding basal glycemia and HbA1c levels, weight, or blood pressure is of high importance. In a study on DM patients from Turkey, herbal supplements such as ginseng, turmeric, thyme, sage, and cinnamon were utilized by 57.6% of the total number of participants [20]. The percentages differ across numerous geographical areas: 58.6% in Saudi Arabia [21], 39.3% in the United Arab Emirates [22], and 62% in Ethiopia [23]. In our online survey, the prevalence of plant supplement use was comparable with the above-described studies at 44.07%.

While numerous factors have been reported to influence the differing percentage of plant supplement use across various studies, female sex has almost universally been identified as a significant contributor [20,21,22,23]. In our study, 68.28% of the responders who utilized plant supplements were female. This finding can be explained by the psychological tendency of women to give more attention to health status, glycemic control, prevention of DM complications, and follow-up visits to medical offices.

Plant supplement type also differs according to geographical area, tradition, beliefs, advertising, and the development of complementary medicine networks [16]. While ginger and cinnamon were the most used plant supplements in Arabia [21], in Turkey, cinnamon, sage, thyme, turmeric and ginseng were predominant [20]. In our study, bitter melon (*Momordica charantia* L.) was by far the most utilized type of plant supplement, followed by blueberry-based supplements and blackseed-based supplements. The high use of *Momordica charantia* L.-based herbal supplements could be explained by the fact that local cultivation of this plant has been increasing for the past 20 years in Romania and many plant supplement manufacturers can include it as a component in supplements designed for DM management [24].

In addition to this reason for the high use of bitter melon among DM patients, the numerous beneficial phytochemicals contained in *Momordica charantia* L. extracts can explain why this plant has imposed itself in the medical literature as an anti-diabetic plant, being among few plants with proof of its beneficial impact in glycemic control from meta-analysis. The several bioactive compounds found in *M. charantia*, including vicine, charantin, glycosides, karavilosides, and polypeptide-p, exhibit insulin-like activity, supporting insulin release and enhancing peripheral glucose uptake [25]. Beyond that, bitter melon has been shown to activate AMP-activated protein kinase (AMPK), a key metabolic enzyme that increases insulin sensitivity and stimulates glucose utilization in muscle and adipose tissues. The plant also inhibits intestinal enzymes like α-glucosidase and α-amylase, slowing carbohydrate digestion and reducing postprandial glucose spikes. In the liver, it downregulates gluconeogenic enzymes such as glucose-6-phosphatase, thereby decreasing endogenous glucose production [26]. The plant’s broad array of phytochemicals, including triterpene, protein, steroids, alkaloids, inorganic, lipid, and phenolic compounds, contributes to its ability to lower glucose absorption, decrease insulin resistance, and improve overall glucose metabolism [27]. Moreover, its rich content of antioxidant and anti-inflammatory phytochemicals contributes to β-cell protection and improved metabolic balance, both of which support more effective glycemic control. These multifaceted mechanisms make *Momordica charantia* L. one of the most promising botanicals in diabetes care, as confirmed by growing clinical and preclinical evidence [26,28].

Plant supplement use was associated in our study with a significantly lower prevalence of diabetic polyneuropathy, an important microvascular complication of DM. Previous studies have reported the contrary: a study performed in Ethiopia identified that those who experienced complications were 1.77 times more likely to be using herbal medicines [29]. The lower rate of diabetic polyneuropathy in our study among plant users could be explained by the fact that they exhibited statistically significantly better glycemic control, given that it is known that hyperglycemia greatly increases the risk of polyneuropathy; however, no causal relationship can be confirmed given the cross-sectional nature of our study.

While other studies have reported that over half of respondents received information on herbal remedies from friends, in our study, supplements were most often recommended by doctors and pharmacists (26.90% and 20%); second most common were patients who took supplements on their own initiative. In a study performed in Saudi Arabia [21], when asked from where they received information on herbal remedies, over half of the respondents (57.4%) said they received it from friends, family, and neighbors. In a study including patients form Ethiopia, 40% took them due to their belief in the advantages of herbal medicine [23], a finding that is somehow similar with our results, where many patients (40.69%) took plant supplements on their own initiative (probably reflecting a personal belief in their benefits). In the study from Saudi Arabia [21], when analyzing the beliefs regarding plant supplement use, diabetes patients most often responded that they use them to prevent the progression of the DM (34.1%) or to lower blood glucose levels (68.2%). These percentages are comparable with the results from our study, where patients used herbal supplements to improve blood glucose levels in 88.97% of cases and to prevent diabetes complications in 27.59% of cases.

The hypothesis of an association between plant supplement use and better glycemic control has been confirmed in our study. This association has been explored in numerous studies using various plant extracts, but the most solid ones remain meta-analyses. In 2019, a meta-analysis was conducted comprising 10 studies (*n* = 1045) on T2DM. Their follow-up period was 4–16 weeks, and their overall risk of bias was moderate to high. A mean difference of −0.72 mmol/L for fasting plasma glucose, −1.43 mmol/L for postprandial plasma glucose, and −0.26% for HbA1c was seen when compared to a placebo in the *Momordica charantia* L. monoherbal formulation [27]. Another meta-analysis including eight trials involving 423 patients with T2DM revealed that supplementing with *Momordica charantia* L. led to significant decreases in fasting blood glucose (−0.85 mmol/L; *p* = 0.005), postprandial glucose (−2.28 mmol/L; *p* = 0.000), and HbA1c (−0.38%; *p* = 0.000) [28]. In our study, patients using plant supplements had statistically significantly lower values of HbA1c and fasting blood glucose compared with non-users, though the level of postprandial glucose was not assessed. It is worth mentioning that patients in our study who were using plant supplements perceived a significant improvement in health in 53.79% of cases, with the greatest proportion reporting lower basal glycemia (70.34%), followed by lower HbA1c (34.48%), and lower postprandial values (28.28%) after using plant supplements. These findings suggest that plant supplement use can contribute to better glycemic control in diabetes patients, which is one of the most important elements in the management of this chronic disease. Few patients mentioned improvements in blood pressure values or weight in our study, a finding that is consistent with meta-analyses, which have not identified any significant improvement in these parameters [28,29].

There are numerous reasons for DM patients not using plant supplements, such as fear of side effects or not being convinced of their benefits [21]. In our research, the main reasons were lack of confidence in their beneficial effects and lack of doctor recommendation. In Ethiopia, the most prevalent motivations for not using herbal supplements were fear of side effects (74.4%) and lack of belief in the benefits of herbal medicine (60.5%) [23].

It is important to highlight the fact that in our study, only 10.34% of the herbal supplement users reported no improvement in their general health after supplement administration. Correlating this result with the fact that 70.34% of plant supplement users reported that they had lower glycemic values in the morning and the fact that average reported HbA1c values in the past 3 months and average reported basal glycemia values were statistically significantly lower compared with non-users, it can be concluded that Romanian patients using plant supplements in the management of their disease are generally satisfied with the outcome and achieve better glycemic control. This finding correlates with numerous studies in the literature where plant supplementation with formulations containing *Momordica charantia* L. [28,29], *Nigella sativa* [30], curcumin [31], or many other herbs led at least to statistically significantly better glycemic control, if not additional improvement in insulin resistance or body weight. All these demonstrate that the role of plant supplements in the management of DM in the Romanian population is not negligible.

To our knowledge, this is the first study in Romania to analyze the prevalence of herbal supplement use in DM patients. Although there are other studies evaluating supplement use in Romanian patients [32,33], no other study has specifically evaluated plant-based supplements and their use among DM patients. Another strong point could be the distribution of the questionnaire among social media diabetes groups, to which patients from all geographical areas of the country have access, therefore increasing the relevance of the results.

Our study has certain limitations. Firstly, it is the cross-sectional study that involved the distribution of a questionnaire via online Facebook DM patient groups. Being conducted online, it could only reach individuals with internet access and basic digital literacy, which may have introduced selection bias. Additionally, the cross-sectional design restricts our ability to draw conclusions about causality between variables. Like any survey that analyses past events (who recommended the plant supplements, what were their initial reasons for using them), there can be recall biases. Furthermore, all clinical and metabolic data, including HbA1c values, glycemia levels, diabetes duration, and complications, were self-reported and could not be independently verified. The absence of direct clinical assessment means that some outcomes may not accurately reflect the true clinical status of participants. Another limitation lies in the identification of herbal remedies. These were reported using vernacular names by patients, without verification or sampling, due to the remote nature of the study. The aim was not to pharmacologically validate supplement content, but rather to capture real-world use and perception. As a result, the precise botanical identity, standardization of active compounds, and potential pharmacological equivalence of the supplements remain unknown, making it impossible to attribute specific glycemic outcomes to individual plant constituents. Future research should focus on well-controlled, multicenter clinical trials, ideally including biochemical validation of supplement content, standardized dosing regimens, and long-term follow-up. It is also essential to explore possible interactions between plant-based supplements and conventional antidiabetic therapies to ensure safe and effective integrated care.

Despite these limitations, our findings offer relevant insight into the real-life behaviors and beliefs of diabetic patients regarding plant-based supplement use. They underscore the need for a structured dialogue between healthcare professionals and patients regarding the responsible and informed integration of herbal therapies in diabetes management.

## 4. Materials and Methods

### 4.1. Study Design and Participants

This was a cross-sectional study conducted using an online questionnaire distributed via Google Forms to diabetes-related social media groups in Romania between February and July 2024. A total of 336 responses were collected, and after validation, 329 questionnaires were deemed eligible for analysis. The inclusion criteria were as follows: participants aged 18 years or older, a confirmed diagnosis of DM, consent to participate in the study, and logical and complete responses to all questions. Questionnaires were excluded if the participants were under 18 years old or provided illogical answers (e.g., reporting unrealistic values for glycated hemoglobin such as 0.5% or providing incorrect names of diabetes medications). Participants were classified into two groups: YES (those using plant supplements) and NO (those not using plant supplements).

The study was approved by the Ethical Committee of the Faculty of Medicine and Pharmacy, University of Oradea (approval number 5/30.10.2023).

### 4.2. Questionnaire Development

The 25-item questionnaire, developed in Romanian, combined open-ended and closed-ended items to capture both structured data and subjective perspectives. It was created through a collaborative process involving three experienced community pharmacists and three physicians specializing in DM, each with over a decade of clinical experience. To ensure accessibility and patient engagement, the questionnaire was written in clear, simple language and included familiar terms for diabetes mellitus patients such as glycemia and HbA1c, enhancing comprehension among respondents with varying levels of health literacy. The instrument was divided into two main sections. The first section, General Information (15 items), was completed by all participants and collected data on socio-demographic characteristics (age, gender, education, and environment), type and duration of diabetes, current drug regimen, diabetes-related complications, and recent clinical indicators such as the most recent HbA1c value (within the past three months) and average morning blood glucose levels.

The second section, Plant Supplement Use (10 items), was directed only at participants who reported using plant-based supplements. Questions addressed the type and form of supplements used (as reported by the patient, using vernacular names), duration and frequency of use, motivation, source of recommendation, and self-perceived impact on health. Non-users were presented with a single, multiple-choice question to determine their reasons for not using such supplements.

The questionnaire was designed with a clear exploratory purpose, to capture how individuals with diabetes actually use plant-based supplements in everyday life, not to determine exact botanical composition, dosage, or preparation methods. Therefore, questions referring to chemical standardization, daily dose, or specific plant parts used (e.g., leaves, fruits, roots) were excluded, as such details could not be verified in an anonymous online survey setting and imply certain botanical science knowledge.

### 4.3. Validation of the Questionnaire

A comprehensive validation process was carried out to ensure the scientific rigor of the instrument, focusing on content validity, construct validity, and criterion validity. The initial version of the questionnaire was reviewed by two pharmacists and two physicians experienced in diabetes management, alongside public health and endocrinology experts. Their feedback led to minor refinements to improve clarity and coherence. In addition, cognitive interviews were conducted with 30 patients diagnosed with diabetes, who were asked to comment on the clarity and relevance of each item. Based on their suggestions, the wording of certain questions was simplified, and the overall flow was improved. Responses from these participants were excluded from the final analysis.

Exploratory factor analysis (EFA) was conducted on grouped items relating to perceived impact of plant supplements on glycemic control (e.g., HbA1c, morning glucose), and motivations for supplement use (e.g., belief in efficacy, dissatisfaction with conventional therapy). The analysis revealed a weak underlying structure, with a Cronbach’s Alpha of 0.38 for the efficacy subscale, indicating limited internal consistency. Factor loadings were also low (<0.2), suggesting that the items may reflect distinct dimensions rather than a unified construct. As such, the items were treated as separate indicators rather than a composite scale.

Statistical comparisons between plant supplement users and non-users revealed that HbA1c levels were significantly lower in users (*p* < 0.001), and fasting (morning) glucose levels were also significantly lower in users (*p* = 0.009). These findings support the criterion-related validity of the questionnaire, indicating that its items effectively differentiate between clinically relevant groups.

The final version of the questionnaire was easy to administer online and well understood by respondents. Despite some limitations in psychometric consistency, the tool proved to be a practical and comprehensive instrument for evaluating socio-demographic factors, supplement use behaviors, and beliefs about plant-based therapies in Romanian patients with diabetes.

### 4.4. Data Analysis

Statistical analysis was conducted using JASP software (version 0.19.2). Descriptive statistics were used to summarize the socio-demographic characteristics of the participants, diabetes-related variables, and plant supplement usage patterns. Continuous variables were reported as medians with interquartile ranges (IQR) due to deviations from normal distribution, as assessed using the Shapiro–Wilk test. For general descriptive purposes, means and standard deviations (SD) were also reported. Categorical variables were expressed as frequencies and percentages. To evaluate group differences between plant supplement users (YES group) and non-users (NO group), we applied the Mann–Whitney U test for continuous variables, as this non-parametric test is appropriate when data do not meet the assumptions of normality. Chi-square (χ^2^) tests were used for categorical comparisons. A *p*-value of less than 0.05 was considered statistically significant. In addition, odds ratios (ORs) with 95% confidence intervals were calculated to evaluate the potential protective effect of plant supplement use on uncontrolled HbA1c (≥6.5%) and elevated fasting glucose (≥100 mg/dL). All included questionnaires were logically validated, and incomplete or inconsistent responses were excluded prior to analysis.

## 5. Conclusions

Our findings suggest that botanical supplements, particularly *Momordica charantia* L., are commonly used by Romanian patients with diabetes and may be associated with better glycemic control. While causality cannot be established due to the study’s design, these insights reflect real-world patient behaviors and perceptions that deserve further exploration. Future research should focus on clinical trials incorporating standardized botanical formulations, clear dosage data, and compositional validation to support the integration of effective plant-derived products into diabetes care. From an industrial perspective, these findings highlight an opportunity for developing evidence-based nutraceuticals tailored to diabetic patients, provided safety, efficacy, and regulatory standards are met.

## Figures and Tables

**Table 1:** Socio-demographic characteristics associated with plant supplement use.

Parameter	YES (*N* = 145)		NO (*N* = 184)		*p* Value
Age (years)	61.62 62	±11.62 [16.0]	58.875 59.5	±13.04 [16.3]	0.068 ^1^
Diabetes duration (years)	8.33 5.0	±7.79 [10.0]	9.745 8.0	±8.03 [11.0]	0.068 ^1^
Living environment					
Urban	95	65.52	108	58.70	0.206 ^2^
Rural	50	34.48	76	41.30	
Sex					
Male	46	31.72	82	44.57	0.018 ^2,^*
Female	99	68.28	102	55.43	
Diabetes type					
Type 1	5	3.45	13	7.07	0.152 ^2^
Type 2	140	96.55	171	92.93	
Schooling					
Medium	82	56.55	122	66.30	0.291 ^2^
Higher education	48	33.10	44	23.91	
Elementary school	14	9.66	17	9.24	
No education	1	0.69	1	0.54	
Working situation					
Retired	69	47.59	79	42,93	0.799 ^2^
Working	71	48.97	96	52,17	
Housewife	3	2.07	6	3,26	
Unemployed	2	1.38	3	1.63	
Treatment					
Biguanides	65	44.83	84	45.65	0.881 ^2^
Sulphonyl urea	21	14.48	18	9.78	0.190 ^2^
SGLT2 inhibitors	25	17.24	26	14.13	0.439^2^
GLP-1agonists	25	17.24	20	10.87	0.090 ^2^
DPP4 inhibitors	10	6.90	9	4.89	0.439 ^2^
SGLT2 + metformin	11	7.59	15	8.15	0.850 ^2^
Biguanides + sulphonyl urea	2	1.38	3	1.63	0.853 ^2^
Biguanides + DPP4	1	0.69	3	1.63	0.439 ^2^
Basal insulin	28	19.31	58	31.52	0.027 ^2,^*
Basal insulin + GLP1	1	0.69	3	1.63	0.439 ^2^
Rapid insulin	8	5.52	19	10.33	0.115 ^2^
Premixed insulin	0	0.00	1	0.54	-
No medication	8	5.52	2	1.09	0.020 *

Values are presented as mean ± SD and median [IQR] for continuous variables due to non-normal distribution. Categorical variables are shown as number (percentage). YES, those using plant supplements; NO, those not using plant supplements; *N*—number of the participants; SD—standard deviation; ^1^ Mann–Whitney U test; ^2^ Chi-square test; * *p* < 0.05.

**Table 2:** Prevalence of blood glucose control according to plant supplement use, *p* = 0.003.

Average Morning Glucose Levels (mg/dL)	YES (*N* = 145)		NO (*N* = 184)		Total
	Number	%	Number	%	Number
<110	30	20.69	37	20.11	67
110–130	60	41.38	47	25.54	107
130–150	33	22.76	51	27.72	84
150–180	19	13.10	30	16.30	49
>180	3	2.07	19	10.33	22
Total	145	100.00	184	100.00	329

YES, those using plant supplements; NO, those not using plant supplements.

**Table 3:** Risk analysis of uncontrolled diabetes and higher than normal basal glycemia.

Plant Supplement Use	YES (*N* = 145)	NO (*N* = 184)	*p* Value
Parameter	%		
HbA1c ≥ 6.5%	71.03	85.87	*p* < 0.001 OR = 0.404 * 95%CI ∈ (0.233; 0.698)
HbA1c < 6.5%	28.97	14.13	
Morning glucose ≥ 100 mg/dL	74.48	83.70	*p* = 0.039 OR = 0.569 * 95%CI ∈ (0.33; 0.976)
Morning glucose < 100 mg/dL	25.52	16.30	

YES, those using plant supplements; NO, those not using plant supplements; OR, odds ratio; * OR < 1, protective factor.

**Table 4:** Distribution of diabetes-specific complications by group.

Plant Supplement Use	YES (*N* = 145)		NO (*N* = 184)		*p* Value
	Number	%	Number	%	
Polyneuropathy					
Yes	45	31.03	78	42.39	0.035 *
No	100	68.97	106	57.61	
Chronic kidney Disease					
Yes	14	9.66	13	7.07	0.395
No	131	90.34	171	92.93	
Retinopathy					
Yes	29	20.00	38	20.65	0.884
No	116	80.00	146	79.35	

YES, those using plant supplements; NO, those not using plant supplements; * *p* < 0.05.

**Table 5:** Type of plant supplement used by the diabetes patients.

What Type of Plant Supplement do You Use?		Number	%
Common Name	Latin Name		
Momordica	*Momordica charantia* L.	51	35.17
Spirulina	*Arthrospira platensis* (Gomont)	2	1.38
Evening primrose	*Oenothera biennis* L.	1	0.69
Plant combination	-	23	15.86
Berberine	*Berberis aristata* DC	6	4.14
Curcumin	*Curcuma longa* L.	9	6.21
Blueberry	*Vaccinium angustifolium* Aiton	22	15.17
Sage	*Salvia officinalis* L.	4	2.76
Resveratrol	-	1	0.69
Mulberry	*Morus* sp.	4	2.76
Dandelion	*Taraxacum officinale* F.H. Wigg.	6	4.14
Blackseed	*Nigella sativa* L.	11	7.59
Blackcurrant	*Ribes nigrum* L.	4	2.76
Cinnamon	*Cinnamomum verum* J.Presl	1	0.69

**Table 6:** Details of plant combination supplements used by the diabetes patients.

Details of Plant Combination (*N* = 23)	Number	%
Momordica, olive extract, fenugreek, cinnamon	1	0.69
Momordica, blueberry	1	0.69
Blueberry, guince	1	0.69
Gymnema, cinnamon	2	1.37
Gymnema, mulberry	1	0.69
Plant supplement with over 30 herbs	1	0.69
Spirulina, sage, mint, rosehip	1	0.69
Momordica, blueberry	1	0.69
Dandelion, bean pods, corn silk	1	0.69
Momordica, gymnema, blueberry, green tea	1	0.69
Curcumin, momordica	1	0.69
Mulberry, blueberry, rosehip	1	0.69
Gingko biloba, blueberry, black seed	1	0.69
Momordica, berberine	1	0.69
Dandelion, berberine	1	0,69
Berberine, blueberry	1	0.69
Momordica, green tea	1	0.69
Mulberry, gymnema	1	0.69
Momordica, mulberry	1	0.69
Momordica, blueberry, guince, walnut	1	0.69
Curcumin, momordica, fenugreek, amla, chirata, gymnema, chinaberry tree, jaman, gurjo	2	1.37

**Table 7:** Type of pharmaceutical form of plant supplements.

What is the Pharmaceutical form of These Supplements?	Number	%
Tea	17	11.72
Capsules	95	65.52
Tablets	26	17.93
Tincture	7	4.83

**Table 8:** Assessment of subjective motives for using plant supplements, dose used, time of use, perceived general benefits.

Parameter	Number	%
Why have you started to use plant supplements to manage your disease?		
To improve blood glucose levels	129	88.97
To prevent diabetes complications	40	27.59
To reduce the risk of cardiovascular disease	18	12.41
To manage diabetes complications	13	8.97
To improve general health	16	11.03
Other	1	0.69
Why do you continue to use plant supplements to manage your disease?		
I trust their effect	101	69.66
I noticed that my blood sugars levels are better	29	20.00
I am not satisfied with classical medication	20	13.79
Friends or family use them, and I follow their example	10	6.90
Other	3	2.07
How long have you been using this supplement?		
>1 year	61	42.07
3–6 months	30	20.69
Less than 3 months	30	20.69
6 months–1 year	24	16.55
This supplement was recommended to you by:		
Doctor	39	26.90
Pharmacist	29	20.00
Mass media	11	7.59
I took it on my own initiative	59	40.69
Personal documentation	1	0.69
Friends	6	4.14
What dose do you use?		
The dose recommended by the doctor/pharmacist	65	44.83
The dose on the leaflet	53	36.55
I don’t have a specific rule regarding the dose I take	27	18.62
What changes have you noticed since using this supplement?		
Significant improvement in health	78	53.79
No improvement	15	10.34
Little improvement in health	52	35.86

**Table 9:** Assessment of specific health benefits after plant supplement use.

Parameters	No.	%
I have lower glycemic values in the morning	102	70.34
I have lower glycemic values after meals	41	28.28
I have lower HbA1c	50	34.48
I lost weight	23	15.86
Blood pressure improved	21	14.48
Cholesterol improved	12	8.28
No parameter improved	15	10.34

**Table 10:** Reasons for not using plant supplements.

Parameters	No.	%
I do not trust their effect	80	43.48
I’m afraid of the side effects	23	12.50
I can’t afford it financially	24	13.04
My doctor didn’t recommend it	70	38.04

## Data Availability

The original contributions presented in this study are included in the article. Further inquiries can be directed to the first authors.

## Abbreviations

The following abbreviations are used in this manuscript:

DM	diabetes mellitus
DPP4	dipeptidyl peptidase 4
EFA	exploratory factor analysis
GLP-1	glucagon-like peptide 1
HbA1c	glycated hemoglobin
SGLT2	sodium-glucose cotransporter 2
